# Chloroplast genome analyses and genomic resource development for epilithic sister genera *Oresitrophe* and *Mukdenia* (Saxifragaceae), using genome skimming data

**DOI:** 10.1186/s12864-018-4633-x

**Published:** 2018-04-04

**Authors:** Luxian Liu, Yuewen Wang, Peizi He, Pan Li, Joongku Lee, Douglas E. Soltis, Chengxin Fu

**Affiliations:** 1 Key Laboratory of Plant Stress Biology, Laboratory of Plant Germplasm and Genetic Engineering, College of Life Sciences, Henan University, Kaifeng, 475000 China; 2 Key Laboratory of Conservation Biology for Endangered Wildlife of the Ministry of Education, and Laboratory of Systematic & Evolutionary Botany and Biodiversity, College of Life Sciences, Zhejiang University, Hangzhou, 310058 China; 3 Department of Environment and Forest Resources, Chungnam National University, Daejeon, 34134 South Korea; 4 Department of Biology, University of Florida, Gainesville, FL 32611 USA

**Keywords:** Chloroplast genome, Cp hotspot, East Asia, Population genetics, SSR

## Abstract

**Background:**

Epilithic sister genera *Oresitrophe* and *Mukdenia* (Saxifragaceae) have an epilithic habitat (rocky slopes) and a parapatric distribution in East Asia, which makes them an ideal model for a more comprehensive understanding of the demographic and divergence history and the influence of climate changes in East Asia. However, the genetic background and resources for these two genera are scarce.

**Results:**

The complete chloroplast (cp) genomes of two *Oresitrophe rupifraga* and one *Mukdenia rossii* individuals were reconstructed and comparative analyses were conducted to examine the evolutionary pattern of chloroplast genomes in Saxifragaceae. The cp genomes ranged from 156,738 bp to 156,960 bp in length and had a typical quadripartite structure with a conserved genome arrangement. Comparative analysis revealed the intron of *rpl*2 has been lost in *Heuchera parviflora*, *Tiarella polyphylla*, *M. rossii* and *O. rupifraga* but presents in the reference genome of *Penthorum chinense*. Seven cp hotspot regions (*trn*H-*psb*A, *trn*R-*atp*A, *atp*I-*rps*2, *rps*2-*rpo*C2, *pet*N-*psb*M, *rps*4-*trn*T and *rpl*33-*rps*18) were identified between *Oresitrophe* and *Mukdenia*, while four hotspots (*trn*Q-*psb*K, *trn*R-*atp*A, *trn*S-*psb*Z and *rpl*33-*rps*18) were identified within *Oresitrophe*. In addition, 24 polymorphic cpSSR loci were found between *Oresitrophe* and *Mukdenia*. Most importantly, we successfully developed 126 intergeneric polymorphic gSSR markers between *Oresitrophe* and *Mukdenia*, as well as 452 intrageneric ones within *Oresitrophe*. Twelve randomly selected intergeneric gSSRs have shown that these two genera exhibit a significant genetic structure.

**Conclusions:**

In this study, we conducted genome skimming for *Oresitrophe rupifraga* and *Mukdenia rossii*. Using these data, we were able to not only assemble their complete chloroplast genomes, but also develop abundant genetic resources (cp hotspots, cpSSRs, polymorphic gSSRs). The genomic patterns and genetic resources presented here will contribute to further studies on population genetics, phylogeny and conservation biology in Saxifragaceae.

**Electronic supplementary material:**

The online version of this article (10.1186/s12864-018-4633-x) contains supplementary material, which is available to authorized users.

## Background

Quaternary climatic oscillations accompanied by glacial and inter-glacial cycles have affected the demographic history of many temperate species, shaped their modern distributions [1, 2], and also left a deep footprint on their genetic structure [3, 4]. East Asia did not develop extensive land ice sheets during the the last glacial maximum (LGM) as Europe and eastern North America did [5]. However, the reduced temperatures (mean reduction = 7–10 °C) and increased aridity have still influenced the distribution and evolution of many plant species in China and neighboring areas [6, 7]. Initially, both paleobotanical and modeling results have revealed that temperate forests in the Northern Hemisphere would have retreated southward (below 30 °N and reaching 25 °N) at the LGM and subsequently recolonized the previously uninhabitable northern regions at the warm and wet interglacial [8–10]. However, recent phylogeographic studies of cool-temperate trees in continental East Asia suggested that, during the LGM, cool-temperate deciduous tree species could have persisted within their modern northern range rather than moving to the south [11–13].

Until recently, there were few independent phylogeographic studies of temperate herbs in East Asia to test these two hypotheses regarding how climatic oscillations affected the range distributions. *Oresitrophe* Bunge and *Mukdenia* Koidz, which are sister genera in Saxifragaceae [14, 15], are both perennial herbs growing on cliffs or rocks. *Oresitrophe* is monotypic, with the only species *O. rupifraga* Bunge occurred in Central and North China [16]; while *Mukdenia* has two species, *M. rossii* (Oliv.) Koidz. and *M. acanthifolia* Nakai, which are distributed from Northeast China to Korean Peninsula [16]. These two sister genera have an epilithic habitat (rocky slopes and ravines) and a parapatric distribution in East Asia, and thus provide an ideal model for a more comprehensive understanding of the demographic and divergence history and the influence of climate changes in East Asia. However, the current studies regarding their genetic background and resources are scarce.

In the last decade, high-throughput sequencing, along with bioinformatic tool development, has provided genomic resources at reasonable prices and schedules [17], with the increasing development of single nucleotide polymorphisms (SNPs) and SSRs in non-model species [18, 19]. In Saxifragaceae, the chloroplast (cp) genome remained relatively unexplored until the release of the only one cp genome of *Heuchera parviflora* (GenBank accession number: KR478645), and these genomic databases were limited to detect and develop the polymorphic markers. More plastid genomes for Saxifragaceae will soon be published as part of the 1KP project [20].

Chloroplast DNA (cpDNA), which is maternally inherited in most angiosperm, usually have a circular structure ranging from 115 to 165 kb in length and contain two copies of a large inverted repeat (IR) region separated by a large single copy (LSC) region and a small single copy (SSC) region [21]. Chloroplast genomes are more conserved than mitochondrial and nuclear genomes in term of gene content, organization and structure [22], and the nucleotide substitution rate of chloroplast genes is at an intermediate level (higher than mitochondria but lower than nuclear) [23]. Considering its small size, conserved gene content and simple structure, the cp genome has been generally applied for understanding the genome evolution, underlying genome size variations, gene and intron losses at higher taxonomic levels [24, 25]. In addition, the non-recombinant nature of plastid genomes and their (generally) uniparental inheritance, makes plastid data a useful tool to trace demographic history, explore species divergence, hybridization and identify species [26, 27]. Traditional screening of cp DNA regions have been chosen mostly based on their efficacy in related taxa for analysis. However, recent studies related on complete chloroplast genome sequences have allowed a more systematic approach to take into account the mutational dynamics of cp genomes [28]. By this method, cp genomic hotspots in terms of informative regions can be identified for a specific plant genus, tribe or family [29, 30]. The conventional technology of Sanger sequencing was time-consuming, troublesome and difficult for reconstructing complete cp genome [31]. In recent years, with the rapid development of high-throughput sequencing technology, especially like Illumina-based genome skimming, more and more complete cp DNA sequences have been isolated and assembled [25, 32]. Subsequently, this has been proven to be a valid and cost-effective to acquire the complete cp DNA and many assembled cp DNA of non-model species have been obtained for the studies such as differential gene expression, genetic markers development [33] and phylogenomics analysis [34].

Simple sequence repeats (SSRs), also called microsatellites containing repetitive sequences of 1–6 bp in length, have been extensively found in both the coding and non-coding sequences of prokaryotic and eukaryotic genomes [35, 36]. Currently, SSRs are broadly applied in various areas of genetic studies including the evaluation of genetic variation [37], construction of genetic linkage maps [38], population genetics [39] and domestication origin of fruit tree species [40, 41], due to their co-dominant inheritance, high polymorphism, reproducibility and transferability. The traditional methods for screening of the polymorphic SSR (polySSR) markers and their subsequent applicability to genetic researches are extremely time-consuming and labor-intensive. However, the recently increasing availability of genome and transcriptome sequences with the decreasing costs of next generation sequencing provides an excellent opportunity and information resources for large-scale mining this type of molecular markers [42]. In recent years, genomic SSR (gSSR) markers have attracted more attention due to detect higher levels of polymorphism relative to EST-SSRs, because intron or intergenic sequences are more variable than extron sequences [43, 44]. Moreover, a series of bioinformatics tools have been developed for automated SSR discovery and marker development, such as CandiSSR or GMATA, which allowed users to identify putative polySSRs not only from the transcriptome datasets but also from multiple assembled genome sequences of a given species or genus along with several comprehensive assessments [42, 45]. It would help researchers to save significant time on marker-screening experiments.

Here, two individuals of *O. rupifraga* and one individual of *M. rossii* were selected for genome skimming. We specifically aimed to: (1) assemble, characterize and compare the cp genomes among representatives of Saxifragaceae in order to gain insights into evolutionary patterns within the family; (2) develop and screen appropriate intergeneric and intrageneric markers (cp hotspot regions, cpSSRs and gSSRs) in *Oresitrophe* and *Mukdenia*.

## Methods

### Plant material, DNA extraction and sequencing

In order to screen polymorphic genomic resources between *Oresitrophe* and *Mukdenia* and within *Oresitrophe*, we selected two individuals of *O. rupifraga* and one individual of *M. rossii* with a long geographical distance, which were theoretically assumed to be more genetically different. Fresh young leaves of two *O. rupifraga* individuals (BJCP: LP161631–1, Muchang, Changping, Beijing, China; HNYD: LP174479–2, Tianmenshan, Zhangjiajie, Hunan, China; Additional file 1: Table S3) and one *M. rossii* (LP174341–20, Taipinghu, Baishan, Jilin, China; Additional file 1: Table S3) were sampled and dried with silica gel. No specific permissions were required for all the samples which are neither privately owned nor protected and the field study did not involve endangered or protected species. The total DNA was extracted using Plant DNAzol Reagent (LifeFeng, Shanghai) according to the manufacturer’s protocol from approximately 2 mg of the silica-dried leaf tissue. The high molecular weight DNA was sheared (yielding ≤800 bp fragments) and the quality of fragmentation was checked on an Agilent Bioanalyzer 2100 (Agilent Technologies). The short-insert (500 bp) paired-end libraries preparation and sequencing were performed by Beijing Genomics Institute (Shenzhen, China). The three samples were pooled with others and run in a single lane of an Illumina HiSeq 2500 with read length of 150 bp.

### Genome assembly and annotation

The raw data was filtered by quality with Phred score < 30 (0.001 probability error) and all remaining high quality sequences were assembled into contigs using the CLC de novo assembler beta 4.06 (CLC Inc., Rarhus, Denmark). The parameters performed in CLC are as follows: deletion and insertion costs of 3, mismatch cost of 2, minimum contig length of 200, bubble size of 98, length fraction and similarity fraction of 0.9. Then, all the contigs were aligned to the reference chloroplast genome (*Heuchera parviflora*) using BLAST (NCBI BLAST v2.2.31) search. The representative chloroplast sequence contigs were ordered and oriented according to the reference chloroplast genome, and the draft chloroplast genome of *O. rupifraga* and *M. rossii* were constructed by connecting overlapping terminal sequences. Finally, clean reads were re-mapped to the draft genome and yielded the complete chloroplast genome sequences.

Initial gene annotation of the three chloroplast genomes was performed through the online program Dual Organellar Genome Annotator [46]. Putative starts, stops, and intron positions were checked according to comparisons with homologous genes of *H. parviflora* cp genome using Geneious v9.0.5 software (Biomatters, Auckland, New Zealand). The tRNA genes were verified with tRNAscan-SE v1.21 [47] with default settings. The circular gene maps were drawn by the OrganellarGenomeDRAW tool (OGDRAW) following by manual modification [48].

### Comparative chloroplast genomic analysis

Multiple complete chloroplast genomes of Saxifragaceae provide an opportunity to compare the sequence variation within the family. Therefore, we included the publicly available chloroplast genome of *Heuchera parviflora*, and *Tiarella polyphylla* (the chloroplast genome has been sequenced by us and will be published soon elsewhere), to compare the overall similarities among different chloroplast genomes in Saxifragaceae, using *Penthorum chinense* (Penthoraceae; JX436155) as the reference based on the results of Dong et al. [24] and Soltis et al. [49]. The sequence identity of the five Saxifragaceae chloroplast genomes was plotted using the mVISTA program with LAGAN mode [50]. The cp DNA rearrangement analyses of five Saxifragaceae chloroplast genomes were performed using Mauve Alignment [51].

### Repeat structure and sequence divergence analysis

We determined the four types of repeat sequences, including direct (forward), inverted (palindromic), complement and reverse repeats in the *Oresitrophe* and *Mukdenia* chloroplast genomes using the online REPuter software with a minimum repeat size of 30 bp and sequence identity greater than 90% [52]. Chloroplast simple sequence repeats (cpSSRs) were detected using Msatcommander v0.8.2 [53] with a threshold ten, five, five, three, three, and three repeat units for mono-, di-, tri-, tetra-, penta-, and hexanucleotide SSRs, respectively.

Multiple alignments of the three sequenced chloroplast genome sequences in this study were carried out using MAFFT version 7.017 [54]. In order to screen variable characters between *Oresitrophe* and *Mukdenia*, the average number of nucleotide differences (K) and total number of mutations (Eta) were determined to analyze nucleotide diversity (Pi) using DnaSP v5.0 [55].

### Polymorphic nucleotide SSR development and validation

Firstly, we removed the chloroplast and mitochondria contigs from the assembled sequences using BLAST (NCBI BLAST v2.2.31) search with the sequence of chloroplast and mitochondria genome of *H. parviflora* (KR478645 & KR559021) as reference. Then, we used CandiSSR [42] to identify candidate polymorphic gSSRs between *Oresitrophe* and *Mukdenia*, as well as within *Oresitrophe*, based on multiple assembled sequences. The parameters performed in CandiSSR are as follows: the flanking sequence length of 100, blast evalue cutoff of 1e-10, blast identity cutoff of 95, blast coverage cutoff of 95. For each target SSRs, primers are automatically designed in the pipeline based on the Primer3 package [56, 57], and global similarities of the primer binding regions is also provided.

Twelve developed intergeneric gSSR markers were randomly selected to test the transferability on 32 individuals (four populations) of *O. rupifraga* and 15 individuals (two populations) of *M. rossii*. Standard PCR amplifications were performed following the conditions below: 94 °C for 1 min; 28 cycles of 94 °C for 30 s, 50–59 °C for 30 s, and 72 °C for 30 s; a final extension at 72 °C for 5 min. Amplification products were checked on 2% agarose gel stained with GeneGreen Nucleic Acid dye (TIANGEN, Beijing, China). Reaction products were subsequently run on an ABI PRISM 3730xl Genetic Analyzer (Applied Biosystems). Genotypes were scored by using the software GeneMarker v2.2.0 (SoftGenetics, LLC, State College, PA, USA). Genetic diversity parameters, including the number of alleles, observed and expected heterozygosity, and polymorphism information content, were estimated using CERVUS v3.0 [58]. Deviations from Hardy-Weinberg equilibrium were tested through GENEPOP v4.2 [59]. SSR genotypes’ assignment to different clusters was tested with STRUCTURE v2.3.3 [60], using 10 replicates of an admixture model allowing for correlated allele frequencies with K ranging from 1 to 10, a burn-in period of 100,000 iterations and a post-burn-in period of 1,000,000 iterations, following recommendations by Gilbert et al,. [61].

## Results

### Genome organization and features

We generated a total of 18,694,896, 15,247,794 and 14,404,890 paired-end (150 bp) clean reads for *O. rupifraga*-BJCP, *O. rupifraga*-HNYD and *M. rossii*, respectively. The de novo assembly generated 352,393 contigs with an N50 length of 346 bp and a total length of 21.69 Mb for *O. rupifraga*-BJCP, 382,827 contigs with an N50 length of 460 bp and a total length of 18.46 Mb for *O. rupifraga*-HNYD, and 352,181 contigs with an N50 length of 397 bp and a total length of 13.64 Mb for *M. rossii*. Each draft chloroplast genome was generated from a combined product of initial contigs (*O. rupifraga*-BJCP: contigs 76, 98, 136, 412 and 1913; *O. rupifraga*-HNYD: contigs 16, 70 and 131; *M. rossii*: contigs 4, 11 and 12), with no gaps and no Ns.

**Fig. 1:**
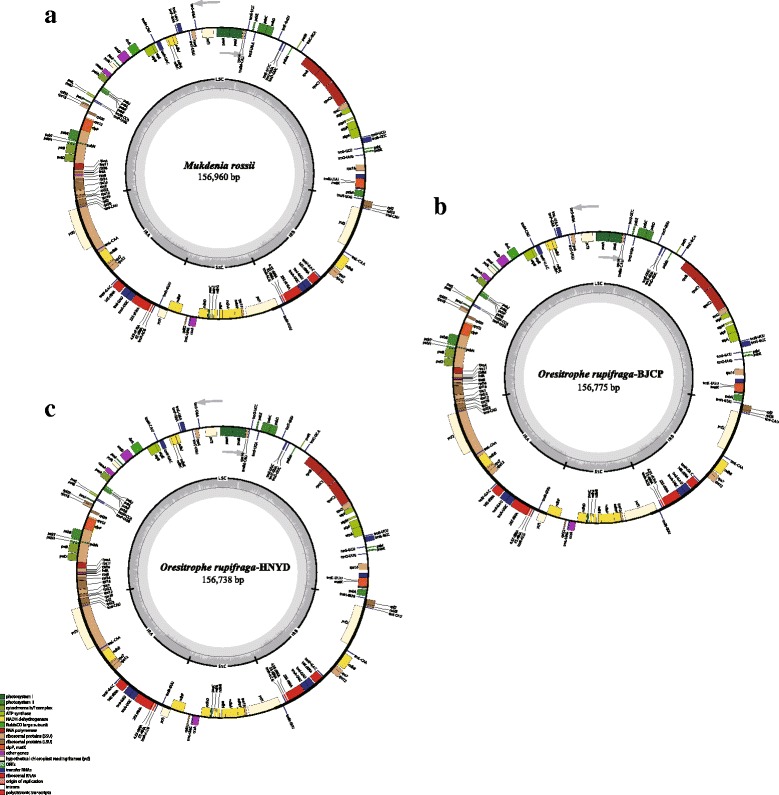
Chloroplast genome maps of *Mukdenia* and *Oresitrophe*: (**a**) *M. rossii*, (**b**) *O. rupifraga*-BJCP and (C) *O. rupifraga*-HNYD. Genes inside the circle are transcribed clockwise, genes outside are transcribed counter-clockwise. The light gray inner circle corresponds to the AT content, the dark gray to the GC content. Genes belonging to different functional groups are shown in different colors

The complete chloroplast genomes of the three samples ranged narrowly from 156,738 bp in *O. rupifraga*-HNYD to 156,960 bp in *M. rossii* (Fig. 1, Table 1). All three chloroplast genomes shared the common feature of comprising two copies of IR (25,507–25,519 bp) separated by the LSC (87,496–87,604 bp) and SSC (18,222–18,342 bp) regions. The overall GC content was 37.80% for *O. rupifraga* and 37.70% for *M. rossii*, whereas the GC content in the LSC, SSC and IR regions were 35.70–35.80, 32.00–32.20 and 43.20%, respectively (Table 1). The chloroplast genome sequences were deposited in GenBank (accession numbers: MF774190 for *O. rupifraga*-BJCP, MG470845 for *O. rupifraga*-HNYD, and MG470844 for *M. rossii*).

**Table 1:** Summary of three chloroplast genomes sequenced in this study

Category	*O. rupifraga*-BJCP	*O. rupifraga*-HNYD	*Mukdenia rossii*
Total cp DNA size (bp)	156,775	156,738	156,960
Length of large single copy (LSC) region (bp)	87,515	87,496	87,604
Length of inverted repeat (IR) region (bp)	25,519	25,509	25,507
Length of small single copy (SSC) region (bp)	18,222	18,224	8342
Coding size (bp)	90,954	90,954	90,954
Intron size (bp)	20,301	20,285	20,290
Spacer size (bp)	45,520	45,499	45,716
Total GC content (%)	37.80	37.80	37.70
GC content of LSC (%)	35.70	35.80	35.70
GC content of IR (%)	43.20	43.20	43.20
GC content of SSC (%)	32.20	32.20	32.00
Total number of genes	113	113	113
Number of protein encoding genes	79	79	79
Number of tRNA genes	30	30	30
Number of rRNA genes	4	4	4
Number of genes duplicated in IR	18	18	18

The three chloroplast genomes encoded an identical set of 131 genes, of which 113 were unique and 18 were duplicated in the IR regions (Tables 1 and 2). The 113 unique genes contained 79 protein-coding genes, 30 tRNA genes, and four rRNA genes. Coding regions, including protein-coding genes, tRNA genes, and rRNA genes, account for 57.95–58.03% of the whole genome, and the remaining regions were non-coding sequences, including inter-genic spacers and introns. Among the 113 unique genes, 14 contain one intron (six tRNA genes and eight protein-coding genes) and three (*rps*12, *clp*P, and *ycf*3) contain two introns. The 5′-end exon of the *rps*12 gene is located in the LSC region, and the intron and 3′-end exon of the gene are situated in the IR region.

**Table 2:** Genes contained in chloroplast genomes (113 genes in total)

Category	Group of gene	Name of gene			
Self-replication	Ribosomal RNA genes	*rrn*4.5^a^	*rrn*5^a^	*rrn*16^a^	*rrn*23^a^
	Transfer RNA genes	*trnA*-UGC^a*^ trnF-GAA *trnH-*GUG *trnL-*CAA^a^ *trnN-*GUU^a^ *trnR-*UCU *trnT-*GGU *trnW-*CCA	*trnC-*GCA *trnfM*-CAU *trnI-*CAU^a^ *trnL-*UAA^*^ trnP-UGG *trnS-*GCU *trnT-*UGU *trnY-*GUA	*trnD-*GUC *trnG-*GCC^*^ trnI-GAU^a*^ trnL-UAG *trnQ-*UUG *trnS-*GGA *trnV-*GAC^a^	*trnE-*UUC *trnG-*UCC *trnK-*UUU^*^ trnM-CAU *trnR-*ACG^a^ *trnS-*UGA *trnV-*UAC^*^
	Small subunit of ribosome	*rps*2 *rps8* *rps*15	*rps*3 *rps*11 *rps*16^*^	*rps*4 *rps*12^a,b**^ rps18	*rps*7^a^ *rps*14 *rps*19
	Large subunit of ribosome	*rpl*2^a^ *rpl*22 *rpl*36	*rpl*14 *rpl*23^a^	*rpl*16^*^ rpl32	*rpl*20 *rpl*33
	RNA polymerase subunits	*rpo*A	*rpo*B	*rpo*C1^*^	*rpo*C2
Photosynthesis	Subunits of photosystem I	*psa*A *psa*J	*psa*B *ycf*3^**^	*psa*C	*psa*I
	Subunits of photosystem II	*psb*A *psb*E *psb*J *psb*N	*psb*B *psb*F *psb*K *psb*T	*psb*C *psb*H *psb*L *psb*Z	*psb*D *psb*I *psb*M
	Subunits of cytochrome	*pet*A *pet*L	*pet*B^*^ petN	*pet*D^*^	*pet*G
	Subunits of ATP synthase	*atp*A *atp*H	*atp*B *atp*I	*atp*E	*atp*F^*^
	Large subunit of Rubisco	*rbc*L			
	Subunits of NADH Dehydrogenase	*ndh*A^*^ ndhE *ndh*I	*ndh*B^a*^ ndhF *ndh*J	*ndh*C *ndh*G *ndh*K	*ndh*D *ndh*H
Other gene	Translational initiation factor	*inf*A			
	Maturase	*mat*K			
	Envelope membrane protein	*cem*A			
	Subunit of acetyl-CoA	*acc*D			
	C-type cytochrome synthesis gene	*ccs*A			
	Protease	*clp*P^**^			
Unknown function	Conserved open reading frames	*ycf*1^a^ (part)	*ycf*2^a^	*ycf*4	

^a^Two gene copies in IRs; ^b^ gene divided into two independent transcription units; one and two asterisks indicate one- and two-intron containing genes, respectively

### Genome comparison of Saxifragaceae

**Fig. 2:**
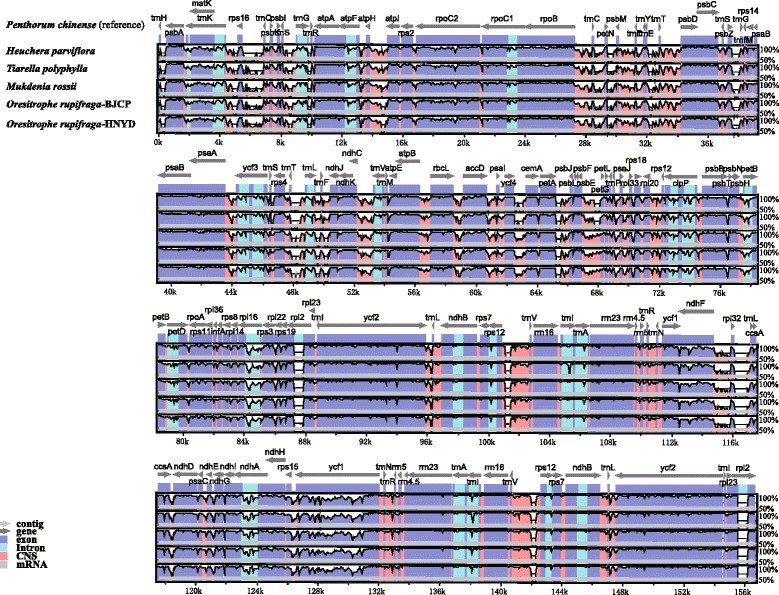
Visualization of alignment of the five Saxifragaceae chloroplast genome sequences, with *Penthorum chinense* as the reference. The horizontal axis indicates the coordinates within the chloroplast genome. The vertical scale indicates the percentage of identity, ranging from 50 to 100%. Genome regions are color coded as protein coding, intron, mRNA, and conserved non-coding sequences (CNS)

**Fig. 3:**
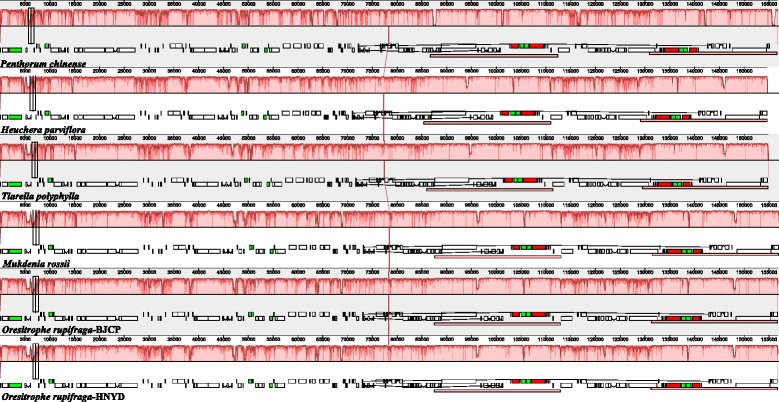
MAUVE alignment of five Saxifragaceae chloroplast genomes. The *Penthorum chinense* genome is shown at top as the reference. Within each of the alignment, local collinear blocks are represented by blocks of the same color connected by lines

The five Saxifragaceae chloroplast genomes were relatively conserved (Fig. 2), and no rearrangement occurred in gene organization after verification (Fig. 3), but differences were still found in terms of genome size, intron losses, and IR expansion and contraction. In addition, the IR region is more conserved in these species than the LSC and SSC regions, which is consistent with other angiosperms [25, 62].

#### Genome size

**Fig. 4:**
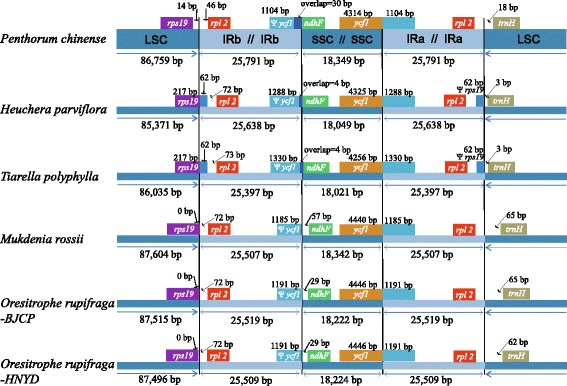
Comparison of the borders of large single-copy (LSC), small single-copy (SSC), and inverted repeat (IR) regions among the five Saxifragaceae chloroplast genomes, with the *Penthorum chinense* genome is shown at top as the reference. The location of two parts of inverted repeat region (IR_A_ and IR_B_) was referred to Fig. 1

In terms of the chloroplast genome size observed among the representative Saxifragaceae species, *M. rossii* and *O. rupifraga* exhibited the similar genome size comparing with the reference genome with ranging from 156,690 bp to 156,960 bp, while *H. parviflora* and *T. polyphylla* had the smaller chloroplast genome comparing with the others (154,696 bp for *H. parviflora* and 154.850 bp for *T. polyphylla*, respectively; Fig. 4).

#### Intron loss

The *rps*16 intron has been lost from the reference genome of *Penthorum chinense*, although it is present in *H. parviflora*, *T. polyphylla*, *M. rossii* and *O. rupifraga*. On the contrary, the *rpl*2 gene in the chloroplast genomes of *H. parviflora*, *T. polyphylla*, *M. rossii* and *O. rupifraga* have lost their only intron except for *P. chinense*.

#### IR expansion and contraction

The expansion and contraction of the border regions between the two IR regions and the single-copy regions will cause the genome size differences among plant lineages. Therefore, we compared the exact IR border positions and their adjacent genes between the five Saxifragaceae chloroplast genomes and the reference genome (Fig. 4). The genes *ycf*1-*ndh*F and *rps*19-*rpl*2-*trn*H were located in the junctions of SSC/IR and LSC/IR regions. The *ycf*1 gene spanned the SSC/IR_A_ region and the pseudogene fragment of ^ψ^*ycf*1 varies from 1104 to 1330 bp. The *ndh*F gene is separated from ^ψ^*ycf*1 by spacers with 29 bp in *O. rupifraga* and 57 bp in *M. rossii* respectively, but shares some nucleotides (from 4 to 30 bp) in other three species. The *rps*19 gene in *H. parviflora* and *T. polyphylla* crossed the LSC/IR_B_ region with 62 bp located at the IR_B_ region, and does not extend to the IR_B_ region in *P. chinense*, *M. rossii* and *O. rupifraga*. The *rpl*2 gene is separated from the LSC/IR_B_ border by a spacer varies from 46 to 135 bp, as well as the *trnH* gene is separated from the IR_A_/LSC border by a spacer varies from 3 to 65 bp.

### Repetitive sequences and hotspot regions in cp genomes

**Fig. 5:**
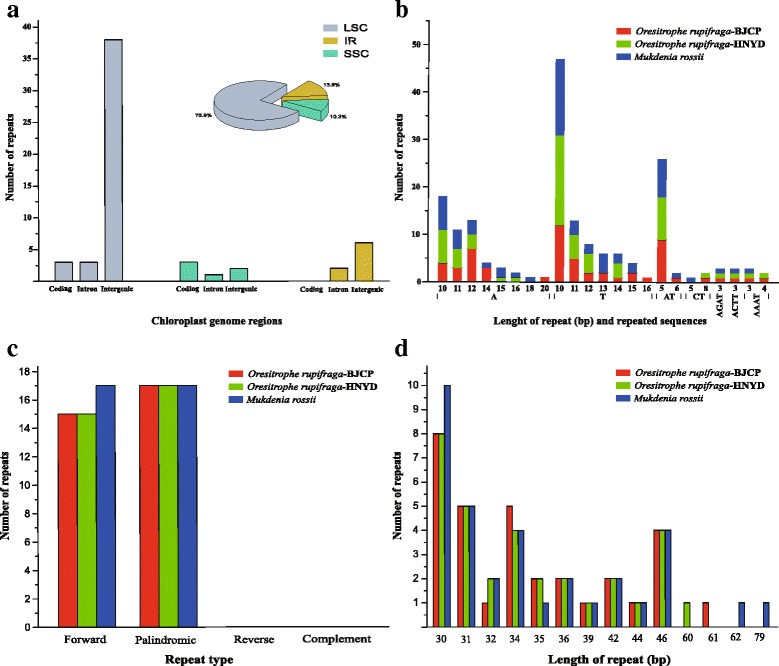
The distribution, type, and presence of simple sequence repeats (SSRs) and analysis of repeated sequences in the cp genome of *Oresitrophe rupifraga* and *Mukdenia rossii*: (**a**) Presence of SSRs in the different region of *O. rupifraga*-BJCP cp genome, (**b**) Presence of polymers in the cp genome of *O. rupifraga* and *M. rossii*, (**c**) Frequency of repeat types, (**d**) Frequency of repeats by length

In the current study, the type, distribution and presence of microsatellites were studied between the cp genomes of *O. rupifraga* and *M. rossii*. A total of 58 perfect microsatellites were identified in the *O. rupifraga*-BJCP cp genome. Among them, 44 were located in the LSC region, whereas 8 and 6 were found in the IR and SSC regions, respectively. Moreover, 6 SSRs were found in the protein-coding regions, 6 were in the introns and 46 were in intergenic spacers of the *O. rupifraga*-BJCP cp genome (Fig. 5a). The distribution and type of microsatellites of other two genomes (*O. rupifraga*-HNYD and *M. rossii*) is shown in supplementary Additional file 2: Figure S1. Among these SSRs, 43 are mononucleotides, 11 are dinucleotides, and 4 are tetranucleotides, tri-, penta-, and hexanucleotides are not found in the cp genomes of *O. rupifraga* and *M. rossii* (Fig. 5b).

In the chloroplast genome of *O. rupifraga* and *M. rossii*, 32 and 34 pairs of repeats (30 bp or longer) were detected using the program REPuter (Kurtz and Schleiermacher, 1999). Among these repeat sequences, 15 and 17 are forward repeats in *O. rupifraga* and *M. rossii* respectively, and the rest of 17 are palindromic repeats in all the three chloroplast genomes (Fig. 5c). In addition, 30–46 bp long repeats occurred in the three chloroplast genomes, as well as 60 bp, 61 bp, 62 and 79 bp long repeats are only detected in *O. rupifraga*-HNYD, *O. rupifraga*-BJCP and *M. rossii* respectively (Fig. 5d).

**Fig. 6:**
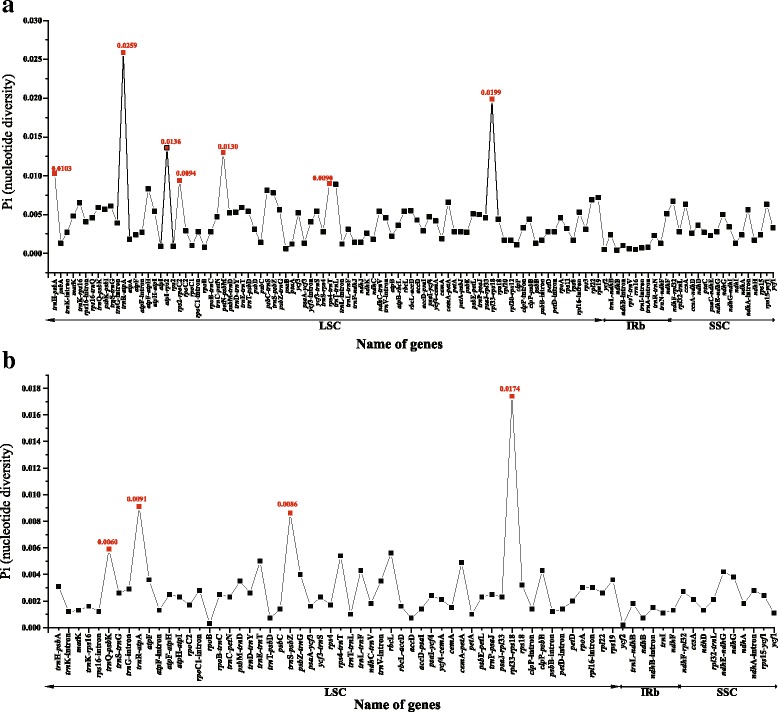
Comparative analysis of the nucleotide variability (*Pi*) values between *Mukdenia rossii* and *Oresitrophe rupifraga* (**a**), and within *O. rupifraga* (**b**)

The coding genes, non-coding regions and intron regions were comparing within *Oresitrophe* and between *Oresitrophe* and *Mukdenia* divergence hotspots. We generated 72 loci (20 coding genes, 40 inter-genic spacers, and 12 intron regions) within *Oresitrophe* and 116 loci (47 coding genes, 53 inter-genic spacers, and 16 intron regions) between *Oresitrophe* and *Mukdenia* with more than 200 bp in length and the nucleotide variability (Pi) values calculated with the DnaSP v5.0 software. Among the values received from comparative analysis, we found it is ranged from 0.0004 (*ndh*B gene) to 0.0259 (*trn*R-*atp*A region) between *Oresitrophe* and *Mukdenia* (Fig. 6a) and from 0.002 (*ycf*2 gene) to 0.0174 (*rpl*33-*rps*18 region) within *Oresitrophe* (Fig. 6b), and the IR region is much more conserved than the LSC and SSC regions. Seven of these variable loci (*Pi* > 0.009) including *trn*H-*psb*A, *trn*R-*atp*A, *atp*I-*rps*2, *rps*2-*rpo*C2, *pet*N-*psb*M, *rps*4-*trn*T and *rpl*33-*rps*18, as well as four variable loci (*Pi* > 0.006) including *trn*Q-*psb*K, *trn*R-*atp*A, *trn*S-*psb*Z and *rpl*33-*rps*18, showed high levels of intergeneric and intrageneric variation.

### Polymorphic genomic SSRs development and validation

**Fig. 7:**
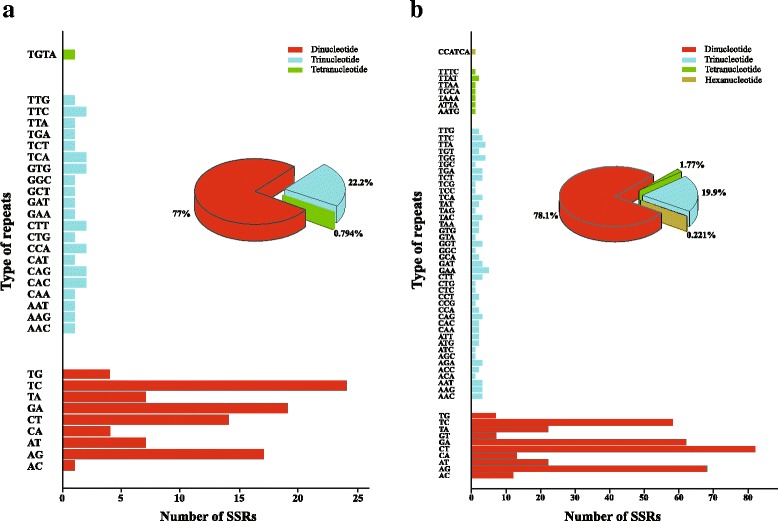
The distribution of polymorphic genomic simple sequence repeats (gSSRs) between *Mukdenia rossii* and *Oresitrophe rupifraga* (**a**), and within *O. rupifraga* (**b**)

A total of 242 candidate polymorphic gSSRs were identified in both *Oresitrophe* and *Mukdenia*. After screening by similarity < 90% (27) and no available primers designed (89), we obtained 126 polymorphic gSSRs with the standard deviation ranged from 0.47 to 4.00 between the two genera (Fig. 7a, Additional file 3: Table S1). Among them, di-, tri-and tetranucleotides account for 77.0%, 22.2% and 0.79%, respectively. In addition, we also detected 691 candidate polymorphic gSSRs within *Oresitrophe*, after removing the loci with the similarity < 90% (31) and no available primers designed (208), we received 452 polymorphic gSSRs with the standard deviation ranged from 0.50 to 5.50, and di-, tri-, tetra- and hexanucleotides account for 78.10%, 19.90%, 1.77% and 0.22%, respectively (Fig. 7b, Additional file 4: Table S2).

To test the transferability of the developed markers, we selected twelve pairs of candidate polySSRs primers (Additional file 3: Table S1) and six populations (Additional file 1: Table S3) including four populations for *O. rupifraga* and two populations for *M. rossii* to detect the effectiveness of primer amplification and to preliminarily assess the genetic variation. Genetic diversity parameters were calculated for two species (Table 3). The polymorphism information content ranged from 0.030 to 0.778, the number of alleles ranged from 2 to 11, and the observed heterozygosity and expected heterozygosity varied from 0.031 to 1.000 and 0.031 to 0.825, respectively. No significant deviation from Hardy-Weinberg equilibrium (*P* < 0.001) was observed for the selected 12 loci except OR242 and OR41 in *O. rupifraga* group, which might be caused by wahlund effect, inbreeding, null alleles and sampling effect.

**Table 3:** The genetic parameters (per locus) in *Oresitrophe rupifraga* and *Mukdenia rossii*

Locus	*Oresitrophe rupifraga* (*N* = 32)				*Mukdenia rossii* (*N* = 15)			
	*A*	*H*_*O*_	*H*_*E*_	PIC^a^	*A*	*H*_*O*_	*H*_*E*_	PIC^a^
OR133	8	0.594	0.800	0.754	6	0.467	0.761	0.696
OR242	2	1.000	0.508	0.375^***^	2	0.867	0.517	0.375
OR9	5	0.375	0.328	0.300	7	0.733	0.818	0.763
OR103	2	0.563	0.411	0.323	3	0.933	0.549	0.421
OR107	6	0.469	0.643	0.566	8	0.800	0.807	0.749
OR127	3	0.094	0.272	0.240	5	0.733	0.634	0.553
OR148	8	0.563	0.743	0.691	7	0.800	0.825	0.772
OR179	11	0.750	0.813	0.778	7	0.933	0.752	0.691
OR212	4	0.188	0.345	0.307	3	0.467	0.559	0.466
OR224	7	0.344	0.568	0.534	4	0.600	0.522	0.458
OR41	7	0.344	0.675	0.624^***^	5	0.600	0.641	0.580
OR131	2	0.031	0.031	0.030	4	0.400	0.531	0.475

*Note*: *A* = number of alleles per locus; *H*_*E*_ = expected heterozygosity; *H*_*O*_ = observed heterozygosity; *N* = number of individuals sampled; *PIC* = polymorphism information content

^a^Significant deviations from Hardy-Weinberg equilibrium at ^*^P < 0.05, ^**^P < 0.01, ^***^P < 0.001, respectively

**Fig. 8:**
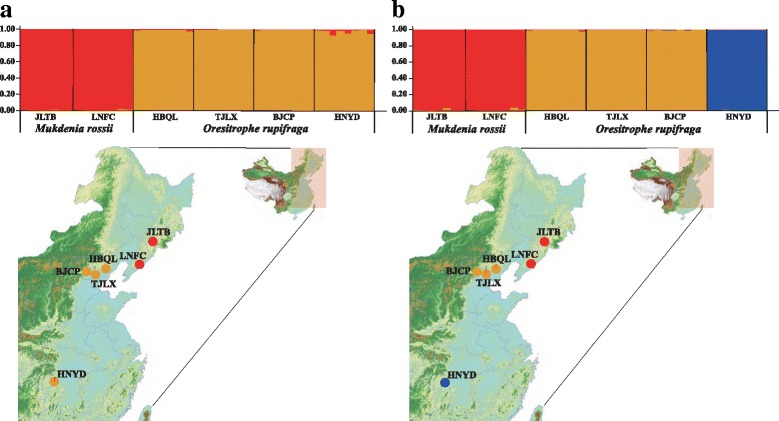
The probability of membership and geographical distribution of gene pools in *Mukdenia rossii* and *Oresitrophe rupifraga*, detected by STRUCTURE analysis: *K* = 2 (**a**) and *K* = 3 (**b**). Each vertical bar represents one individual (*N* = 47), with populations arranged by collection site from Northeast to Central China

In the STRUCTURE analysis, the true number of clusters *K* in the data were difficult to determine following Falush et al. [63], due to ln *P(D)* increased progressively as *K* increased (Additional file 5: Figure S2). The Δ*K* statistic of Evanno et al. [64], however, permitted detection of a rate change in ln *P(D)* corresponding to *K* = 2. At *K* = 2, all the six populations were separated into two clusters according to the different species (Fig. 8a). Moreover, for *K* = 3, we found that four *O. rupifraga* populations were further separated into two clusters, with HBQL, TJLX and BJCP assigned into one cluster, and HNYD into the second cluster (Fig. 8b).

## Discussion

### Chloroplast genome organization of *Oresitrophe* and *Mukdenia* and genome evolution in Saxifragaceae

The availability of plastid genome sequences for most major lineages of angiosperms has increased rapidly with next generation sequencing (NGS) methods development during the past decade. These data have provided many new insights into angiosperm phylogenetic relationships [25, 65], genomic rearrangements [66, 67], and genome-wide patterns and rates of nucleotide substitutions [68, 69]. In Saxifragaceae, the chloroplast genomes remained relatively limited, with only one species (*Heuchera parviflora*) was sequenced. In this study, we assembled and annotated three complete chloroplast genomes including two of *Oresitrophe rupifraga* and one of *Mukdenia rossii*. By comparing cp genomes in Saxifragaceae, we were able to gain s insights into evolutionary patterns of the family.

Comparative analyses of three chloroplast genomes sequenced in this study also showed highly conserved structures and genes. The size of *O. rupifraga*-BJCP, *O. rupifraga*-HNYD, and *M. rossii* ranged narrowly from 156,738 bp to 156,960 bp with sharing the common feature of comprising two copies of IR separated by the LSC and SSC regions. Most angiosperms commonly encode 74 protein-coding genes, while an additional five are present in only some species [70]. However, the three cp genomes contained 79 protein-coding genes, 30 tRNA genes, and four rRNA genes, which is similar to *Heuchera parviflora* and *Penthorum chinense*. This might have been because the genome shares its gene contents with the Saxifragaceae family.

After comparing the cp genomes between four Saxifragaceae species and the reference, we found the gene content and genome structure were relatively conserved, and no rearrangement occurred in gene organization, but some differences were detected in terms of intron losses and IR expansion and contraction. Two genes, *rpl*2 and *rps*16, presented the intron loss phenomenon. The *rpl*2 intron loss has been reported in some Saxifragaceae genera, such as *Saxifraga* and *Heuchera* [71]. This phenomenon was subsequently confirmed in *Heuchera sanguinea* (HQ664603), but was absent in *H. micrantha* (EF207446) and *Saxifraga stolonifera* (EF207457). In this study, the *rpl*2 intron is lost in all four representative species, suggesting that intron loss in the *rpl*2 gene is not occasional in Saxifragaceae. The *rps*16 gene has lost its only intron in the chloroplast genome of *Penthorum chinense*, but present in *Oresitrophe rupifraga*, *Mukdenia rossii*, *Heuchera parviflora* and *Tiarella polyphylla*, which is similar to those of the other published Saxifragales species [24]. Previously studies have reported the *rps*16 intron loss is also detected in *Trachelium* (Campanulaceae) [67] and *Pelargonium* (Geraniaceae) [72], we still deduced this phenomenon is unusual in normal angiospermous chloroplast genomes because the genome of *Trachelium* and *Pelargonium* have been extensively restructured. Moreover, the *ycf*15 gene, which displays a small open reading frame (ORF) with potential function in tobacco [73], was pseudogenized in all five representatives of Saxifragaceae. The *inf*A gene, which functions as a translation initiation factor [74] with loss of it having independently experienced multiple times during the evolution of land plants [70], appears in all of the species in this study. Thus, we inferred that the pseudogenization of *ycf*15 and attendant of *inf*A are ancestral condition in Saxifragaceae.

The border regions of LSC/IR_B_, IR_B_/SSC, SSC/IR_A_, and IR_A_/LSC represent highly variable regions with many nucleotide changes in cp genomes of closely related species [75]. Therefore, we compared the exact IR border positions and their adjacent genes among the five Saxifragaceae chloroplast genomes and the reference genome. The result showed that *T. polyphylla* and *H. parviflora* have relatively similar boundary characteristics with the *rps*19 gene locating at the junction of LSC/IR_B_ region of cp genome and the *ndh*F gene sharing some nucleotides with the *ycf*1 pseudogene. Whereas *M. rossii* and *O. rupifraga* presented similar boundary characteristics with the *rps*19 gene does not extending to the IR_B_ region and the *ndh*F gene does not sharing any nucleotides with the *ycf*1 pseudogene. The reference genome of *P. chinense* showed a relatively independent boundary feature comparing with the Saxifragaceae species. In Saxifragaceae, we deduced that the species with closer phylogenetic relationship will have more similar boundary feature. However, due to limited species were sampled, we need more chloroplast genome sequences to test our hypothesis in the future.

### Molecular markers development using genome skimming

*Oresitrophe* and *Mukdenia* provide an ideal model for a more comprehensive understanding of the divergence history and the influence of climate changes on lithophytes in Northeast China and adjacent regions. However, no genetic background and resources are available for these two genera. By analyzing genome skimming data of *Oresitrophe* and *Mukdenia*, here we developed abundant genetic resources, including cp hotspot regions, cpSSRs and polymorphic gSSRs.

Mutation events in the chloroplast genome are usually clustered in “hotspots”, and these mutational dynamics created highly variable regions dispersed throughout the chloroplast genomes [76, 77]. We identified seven regions including *trn*H-*psb*A, *trn*R-*atp*A, *atp*I-*rps*2, *rps*2-*rpo*C2, *pet*N-*psb*M, *rps*4-*trn*T and *rpl*33-*rps*18 between *Oresitrophe* and *Mukdenia*, as well as four highly variable regions including *trn*Q-*psb*K, *trn*R-*atp*A, *trn*S-*psb*Z and *rpl*33-*rps*18 within *Oresitrophe*, which enabled the development of novel cp markers for genetic studies in these two genera. As our results showed, all of them occurred in the LSC region but not in SSC or IR regions. Among these regions, the highly variable regions *trn*H-*psb*A, *atp*I-*rps*2, *pet*N-*psb*M and *rpl*33-*rps*18 have been reported in seed plants before [25, 78–81]. The hotspot regions will provide important genetic information for the subsequent studies on phylogeography and divergence history of *Oresitrophe* and *Mukdenia*.

Chloroplast simple sequence repeats (cpSSRs) markers, which possess unique and important characteristics such as non-recombination, haploidy, uniparental inheritance and a low nucleotide substitution rate, are excellent tool in population genetics [82]. Particularly, the chloroplast genome holds ancient genetic patterns and can therefore provide unique insight into evolutionary processes [83], and cpSSR loci are generally distributed throughout noncoding regions with higher sequence variations than coding regions [84]. Moreover, the cpSSR markers developed based on a species are frequently universal to amplify homologous loci across related taxa [85]. Thus, cpSSR markers can be used to reveal population genetic variation and phylogeographic patterns [86, 87]. In this study, the type, distribution and presence of cpSSRs were detected between the chloroplast genomes of *O. rupifraga* and *M. rossii*. We received a lot of 58, 61 and 61 perfect cpSSR loci in *O. rupifraga*-BJCP, *O. rupifraga*-HNYD and *Mukdenia rossii*, respectively. After comparative analysis, 24 polymorphic cpSSR loci were developed between *Oresitrophe* and *Mukdenia* (Additional file 6: Table S4), which will contribute to further researches relating on population genetic and phylogeography of these two genera.

With the application of the NGS technologies, genomic resources have greatly increased in the last decade [88]. Recently, the increasing of available whole-genome or transcriptome sequences has provided considerable resources for SSR mining and SSR marker applications for research and genetic improvements [89]. A series of bioinformatics tools for SSRs have also been developed, such as MISA [90], SSR Primer [91], and SSR Locator [92]. However, these tools have not yet integrated a computational solution for systematic assessment of SSR polymorphic status, thus the detected SSRs still require manual screening for the polymorphy [45]. CandiSSR is a new pipeline to detect candidate polymorphic SSRs not only from the transcriptome datasets but also from multiple assembled genome sequences [42].

In this study, we employed genome skimming data not only to complete the plastid genome assembly of *O. rupifraga* and *M. rossii*, but also to identify appropriate intergeneric and intrageneric polymorphic gSSRs using CandiSSR. Some of these markers may have wide utility in Saxifragaceae, a family that with other Saxifragales has provided a useful well-sampled model for the study of niche evolution and ecological diversification [93]. We developed 126 and 452 intergeneric and intrageneric polySSR markers between *Oresitrophe* and *Mukdenia* and within *Oresitrophe*. Twelve pairs of candidate gSSR primers were selected to test their transferability following Qi et al. [94], primer transferability was detected using 2% agarose gels, and amplification was considered successful when one clear distinct band was visible in the expected size range. In total, 100% of the developed microsatellite markers we selected could be successfully amplified in two populations of *M. rossii* and four populations of *O. rupifraga*. Genetic diversity parameters initially indicated *M. rossii* (*H*_*E*_ = 0.66) and *O. rupifraga* (*H*_*E*_ = 0.51) have a pattern of moderate genetic diversity, and the genetic diversity observed in *M. rossii* is very similar to the average *H*_*E*_ of 0.65 for outcrossing plant species from other microsatellite studies [39, 95]. STRUCTURE analysis separated the six populations into two clusters according to the different species at *K* = 2, and *O. rupifraga* populations were further assigned to two distinct clusters at *K* = 3, preliminarily showing that the two close genera have relatively significant geographical structure. In the near future, we will expand our sampling of *Oresitrophe* and *Mukdenia* to study the population genetic structure and phylogeography of these two genera.

## Conclusions

In present study, we conducted genome skimming for *Oresitrophe* and *Mukdenia*. Using these data, we assembled their complete chloroplast genomes and developed abundant genetic resources including cp hotspots, cpSSRs and polymorphic gSSRs. The cp genomes had a typical quadripartite structure with a conserved genome arrangement, and the evolutionary pattern of cp genomes in Saxifragaceae was also examined utilizing four representative genera. In addition, the intergeneric gSSRs we randomly selected have shown that *Oresitrophe* and *Mukdenia* exhibited a significant genetic structure. The genomic patterns and genetic resources presented in this study will contribute to further studies on population genetic, phylogeny and conservation biology in Saxifragaceae.

## Additional files

Additional file 1: **Table S3.** Locality and voucher information for populations of *Oresitrophe rupifraga* and *Mukdenia rossii* used in this study. Voucher specimens are deposited at the herbarium of Zhejiang University (HZU), Hangzhou, Zhejiang, China. (DOCX 17 kb)

Additional file 2: **Figure S1.** The distribution and presence of simple sequence repeats (SSRs) in the cp genome of *Oresitrophe rupifraga*-HNYD (A) and *Mukdenia rossii* (B). (PDF 392 kb)

Additional file 3: **Table S1.** The detail information of polymorphic gSSRs identified within *Oresitrophe* and between *Oresitrophe* and *Mukdenia*. (XLSX 103 kb)

Additional file 4: **Table S2.** Primer pairs designed for each detected polymorphic gSSRs within *Oresitrophe* and between *Oresitrophe* and *Mukdenia*. (XLSX 157 kb)

Additional file 5: **Figure S2.** Summary of STRUCTURE analyses based on the gSSR data. (A) Mean ln posterior probabilities of each *K*, LnP(D). (B) The corresponding Δ*K* statistics calculated according to Evanno et al. (2005). (PDF 354 kb)

Additional file 6: **Table S4.** cpSSRs identified from comparative analysis of chloroplast genome for *Oresitrophe* and *Mukdenia*. (DOCX 22 kb)
