# Supplementary material for: Chloroplast genome analyses and genomic resource development for epilithic sister genera Oresitrophe and Mukdenia (Saxifragaceae), using genome skimming data
Source: BMC Genomics. 2018 Apr 4;19:235. doi: 10.1186/s12864-018-4633-x (PMC5885378; doi:10.1186/s12864-018-4633-x)
Supplement: Supplementary file 1 — Table S3. Locality and voucher information for populations of Oresitrophe rupifraga and Mukdenia rossii used in this study. Voucher specimens are deposited at the herbarium of Zhejiang University (HZU), Hangzhou, Zhejiang, China. (DOCX 17 kb) [file 12864_2018_4633_MOESM1_ESM.docx]

Table S3. Locality and voucher information for populations of *O. rupifraga* and *M. rossii* used in this study. Voucher specimens are deposited at the herbarium of Zhejiang University (HZU), Hangzhou, Zhejiang, China.

| Species | Population code | Voucher no. | Locality | Geographic coordinates | Altitude (m) | n |
| --- | --- | --- | --- | --- | --- | --- |
| *Oresitrophe rupifraga* | HBQL | LP161448 | Qinglong, Hebei, China | 40°34’53.12" N, 119°07’20.58" E | 261 | 8 |
|  | TJLX | LP161522 | Lixian, Tianjin, China | 40°07’54.81" N, 117°30’32.46" E | 87 | 8 |
|  | BJCP | LP161631 | Changping, Beijing, China | 40°18’18.77" N, 116°24’31.21" E | 273 | 8 |
|  | HNYD | LP174479 | Zhangjiajie, Hunan, China | 29°03’02.66" N, 110°28’58.15" E | 268 | 8 |
| *Mukdenia rossii* | JLTB | LP174341 | Baishan, Jilin, China | 41°54’24.53" N, 126°18’09.82" E | 483 | 8 |
|  | LNFC | LP174393 | Fengcheng, Liaoning, China | 40°25’08.29" N, 124°04’36.97" E | 292 | 8 |
