# Supplementary figures and images for: Chloroplast genome analyses and genomic resource development for epilithic sister genera Oresitrophe and Mukdenia (Saxifragaceae), using genome skimming data
Source: BMC Genomics. 2018 Apr 4;19:235. doi: 10.1186/s12864-018-4633-x (PMC5885378; doi:10.1186/s12864-018-4633-x)

**A**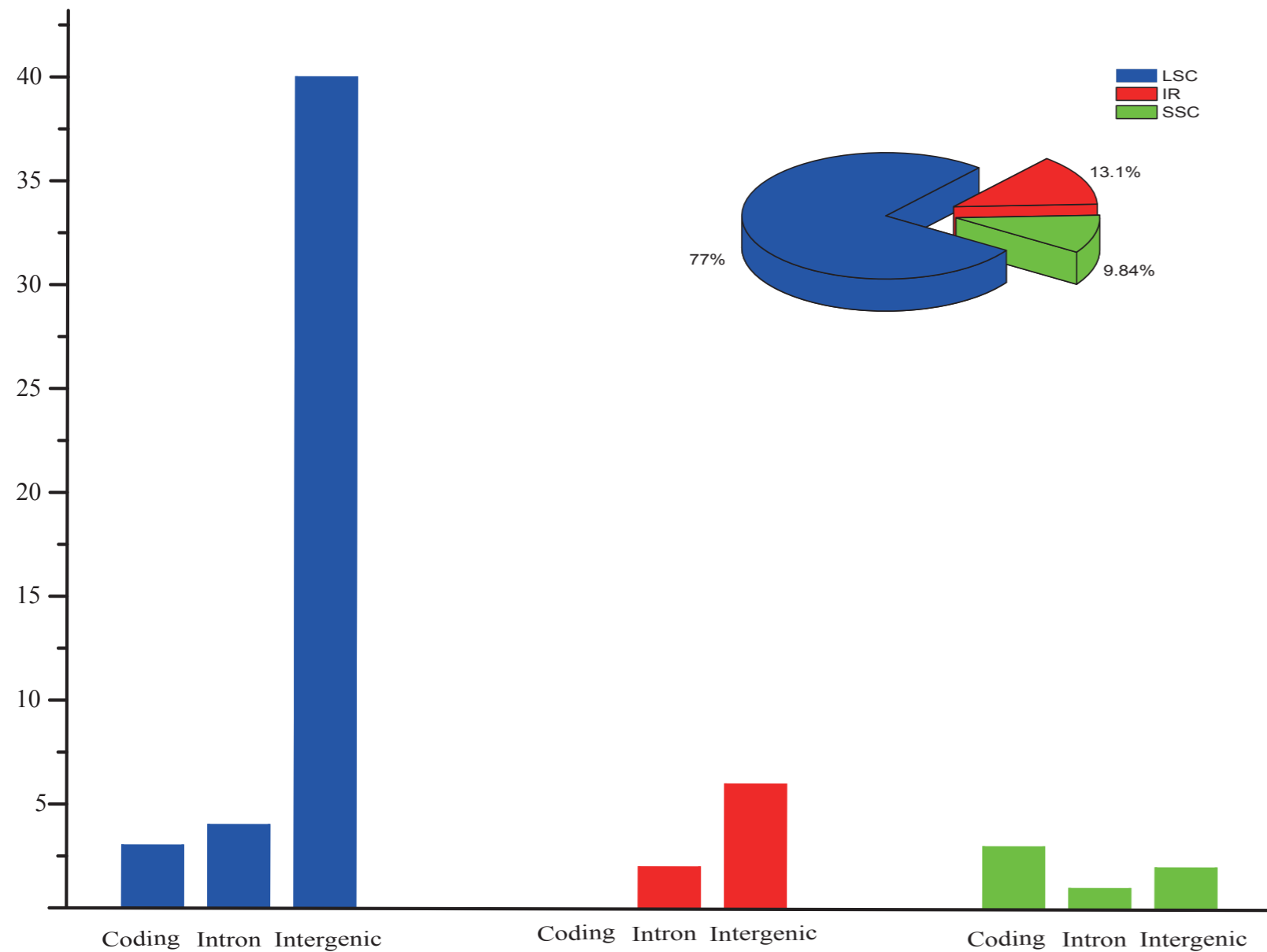**B**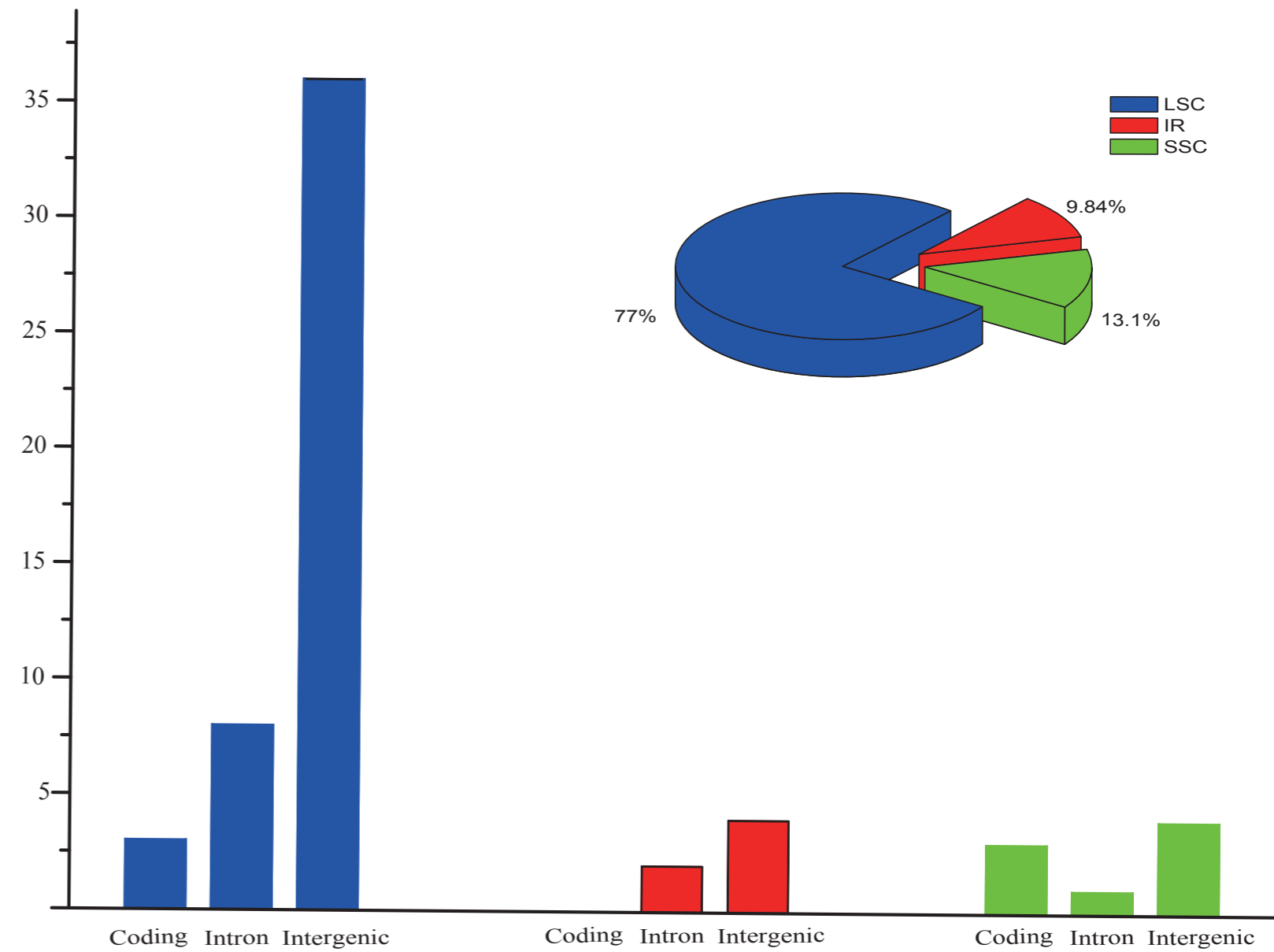

Supplement: Supplementary file 2 — Figure S1. The distribution and presence of simple sequence repeats (SSRs) in the cp genome of Oresitrophe rupifraga-HNYD (A) and Mukdenia rossii (B). (PDF 392 kb) [file 12864_2018_4633_MOESM2_ESM.pdf]

A

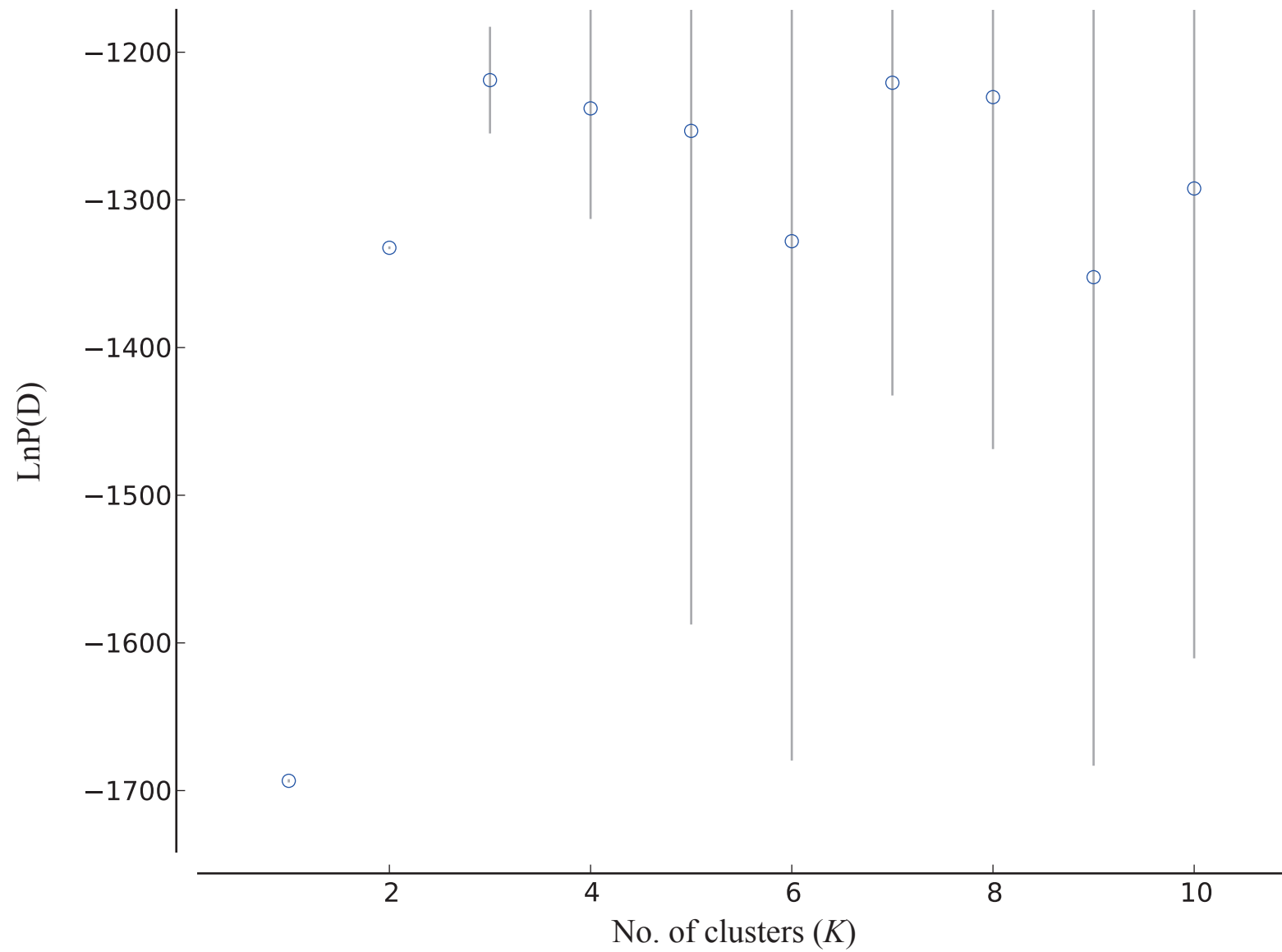

B

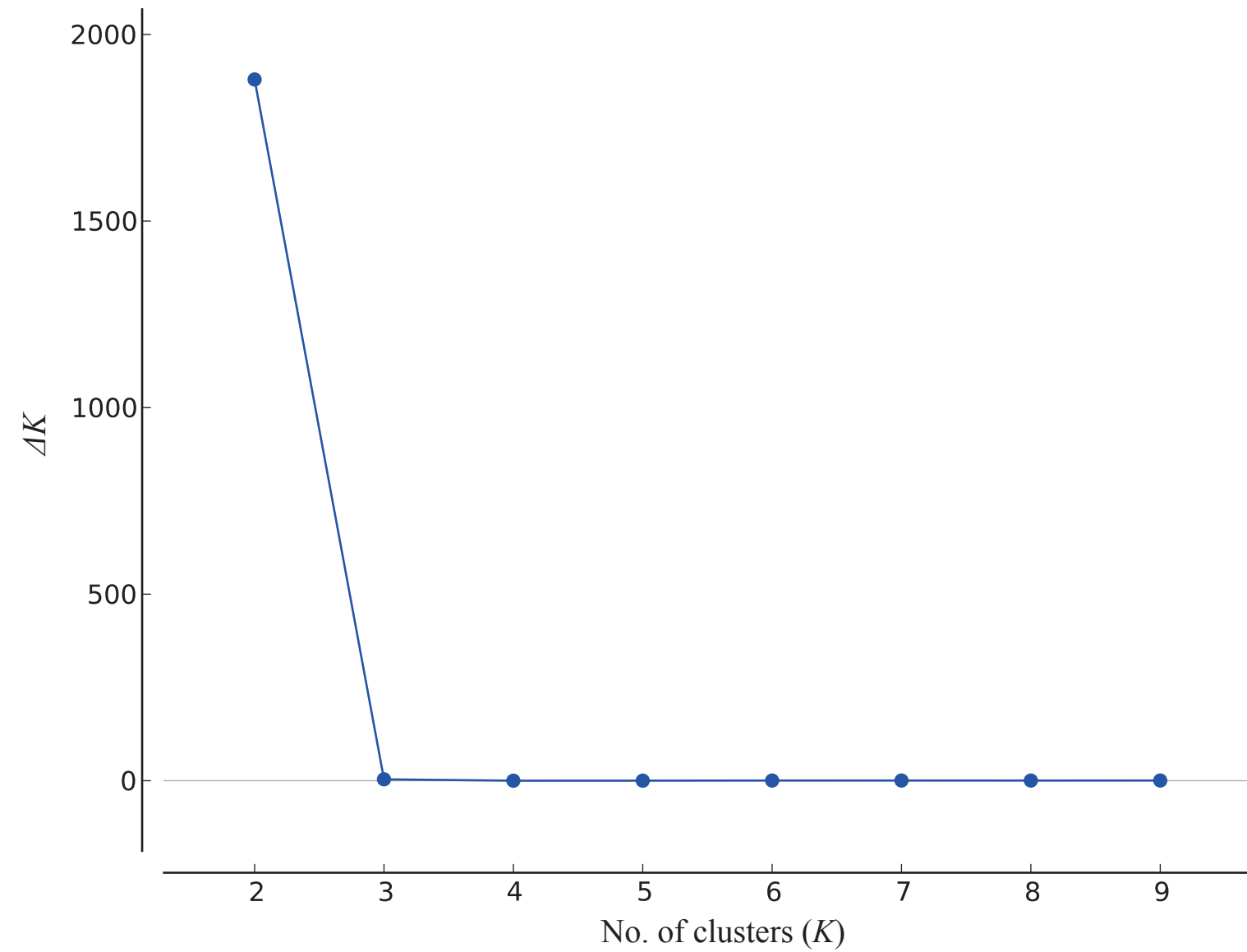

Supplement: Supplementary file 5 — Figure S2. Summary of STRUCTURE analyses based on the gSSR data. (A) Mean ln posterior probabilities of each K, LnP(D). (B) The corresponding ΔK statistics calculated according to Evanno et al. (2005). (PDF 354 kb) [file 12864_2018_4633_MOESM5_ESM.pdf]
