# Supplementary material for: Chloroplast genome analyses and genomic resource development for epilithic sister genera Oresitrophe and Mukdenia (Saxifragaceae), using genome skimming data
Source: BMC Genomics. 2018 Apr 4;19:235. doi: 10.1186/s12864-018-4633-x (PMC5885378; doi:10.1186/s12864-018-4633-x)
Supplement: Supplementary file 6 — Table S4. cpSSRs identified from comparative analysis of chloroplast genome for Oresitrophe and Mukdenia. (DOCX 22 kb) [file 12864_2018_4633_MOESM6_ESM.docx]

| No. | Position | Species | Start | End | SSR type | Species | Start | End | SSR type | Species | Start | End | SSR type |
| --- | --- | --- | --- | --- | --- | --- | --- | --- | --- | --- | --- | --- | --- |
|  |  | *O. rupifraga*  -BJCP |  |  |  | *O. rupifraga*  -HNYD |  |  |  | *M. rossii* |  |  |  |
| 1 | *trn*S-*trn*G |  | 9453 | 9465 | (A)12 |  | 9455 | 9465 | (A)10 |  | 9430 | 9445 | (A)15 |
| 2 | *trn*G-*trn*R |  | 10585 | 10595 | (T)10 |  | 10567 | 10577 | (T)10 |  | 10536 | 10550 | (T)14 |
| 3 | *trn*R-*atp*A |  | 10989 | 11001 | (T)12 |  | 10963 | 10975 | (T)12 |  | 10917 | 10932 | (T)15 |
| 4 | *atp*A-*atp*F |  | 12603 | 12623 | (A)20 |  | 12577 | 12593 | (A)16 |  | 12529 | 12545 | (A)16 |
| 5 | *atp*F-*atp*H |  | 14004 | 14019 | (T)15 |  | 13974 | 13988 | (T)14 |  | 13918 | 13929 | (T)11 |
| 6 | *atp*H-*atp*I |  | 14605 | 14619 | (A)14 |  | 14577 | 14592 | (A)15 |  | 14521 | 14531 | (A)10 |
| 7 | *rpo*B-*trn*C |  | 28617 | 28628 | (T)11 |  | 28590 | 28601 | (T)11 |  | 28767 | 28777 | (T)10 |
| 8 | *pet*N-*psb*M |  | 30144 | 30155 | (T)11 |  | 30117 | 30127 | (T)10 |  | 30288 | 30301 | (T)13 |
| 9 | *rps*14-*psa*B |  | 39693 | 39707 | (A)14 |  | 39654 | 39666 | (A)12 |  | 39793 | 39811 | (A)18 |
| 10 | *ycf*3-*trn*S |  | 47502 | 47514 | (A)12 |  | 47461 | 47472 | (A)11 |  | 47626 | 47638 | (A)12 |
| 11 | *atp*B-*rbc*L |  | 57863 | 57875 | (A)12 |  | 57843 | 57855 | (A)12 |  | 57986 | 57997 | (A)11 |
| 12 | *pet*A-*psb*J |  | 66498 | 66508 | (T)10 |  | 66487 | 66497 | (T)10 |  | 66578 | 66591 | (T)13 |
| 13 | *psb*E-*pet*L |  | 68753 | 68765 | (A)12 |  | 68744 | 68755 | (A)11 |  | 68837 | 68849 | (A)12 |
| 14 | *pet*G-*trn*W |  | 69538 | 69551 | (T)13 |  | 69528 | 69540 | (T)12 |  | 69622 | 69633 | (T)11 |
| 15 | *rps*18-*rpl*20 |  | 71723 | 71737 | (A)14 |  | 71712 | 71724 | (A)12 |  | 71807 | 71818 | (A)11 |
| 16 | *rp1*20-*rps*1*2* |  | 72480 | 72492 | (A)12 |  | 72467 | 72477 | (A)10 |  | 72560 | 72571 | (A)11 |
| 17 | *clp*P intron |  | 74018 | 74030 | (A)12 |  | 74823 | 74833 | (T)10 |  | 74108 | 74118 | (A)10 |
| 18 | *inf*A-*rps*8 |  | 83346 | 83356 | (T)10 |  | 83332 | 83342 | (T)10 |  | 83431 | 83444 | (T)13 |
| 19 | *rpl*14-*rpl*16 |  | 84391 | 84402 | (A)11 |  | 84377 | 84388 | (A)11 |  | 84481 | 84495 | (A)14 |
| 20 | *rpl*16 intron |  | 85110 | 85125 | (T)15 |  | 85096 | 85108 | (T)12 |  | 85203 | 85213 | (T)10 |
| 21 | *rpl*16 intron |  | 85814 | 85830 | (T)16 |  | 85797 | 85811 | (T)14 |  | 85902 | 85917 | (T)15 |
| 22 | *rrn*5-*trn*R |  | 110579 | 110590 | (A)11 |  | 110552 | 110562 | (A)10 |  | 110665 | 110677 | (A)12 |
| 23 | *ndh*A intron |  | 123610 | 123626 | (CT)8 |  | 123584 | 123600 | (CT)8 |  | 123745 | 123755 | (CT)5 |
| 24 | *ycf*1 |  | 130060 | 130070 | (T)10 |  | 130033 | 130043 | (T)10 |  | 130263 | 130275 | (T)12 |
